# Characterization of mesocolic adipose hyperplasia in a rat 2,4,6-trinitrobenzenesulfonic acid colitis model and comparison to creeping fat in Crohn disease

**DOI:** 10.1093/ibd/izaf328

**Published:** 2026-02-18

**Authors:** Laura Clua-Ferré, Roger Suau, Montserrat Guasch, Diandra Monfort-Ferré, Albert Boronat-Toscano, Micaella Aquino, Karol Matute-Molina, Mireya Jimeno, Míriam Mañosa, Lauro Sumoy, Eugeni Domènech, Ramon Bartolí, Josep Manyé, Carolina Serena

**Affiliations:** Inflammatory Bowel Diseases Research Group, Germans Trias i Pujol Research Institute (IGTP), Badalona, Spain; University Hospital Joan XXIII, Pere Virgili Health Research Institute (IISPV), Rovira i Virgili University, Tarragona, Spain; Inflammatory Bowel Diseases Research Group, Germans Trias i Pujol Research Institute (IGTP), Badalona, Spain; Inflammatory Bowel Diseases Research Group, Germans Trias i Pujol Research Institute (IGTP), Badalona, Spain; Department of General and Digestive Surgery, Hospital de Viladecans, Viladecans, Spain; University Hospital Joan XXIII, Pere Virgili Health Research Institute (IISPV), Rovira i Virgili University, Tarragona, Spain; University Hospital Joan XXIII, Pere Virgili Health Research Institute (IISPV), Rovira i Virgili University, Tarragona, Spain; Department of Pathology, Germans Trias i Pujol University Hospital, Badalona, Spain; Department of Pathology, Germans Trias i Pujol University Hospital, Badalona, Spain; Department of Pathology, Germans Trias i Pujol University Hospital, Badalona, Spain; Inflammatory Bowel Diseases Research Group, Germans Trias i Pujol Research Institute (IGTP), Badalona, Spain; Biomedical Research Network Center for Hepatic and Digestive Diseases (CIBEREHD), Badalona, Spain; Department of Gastroenterology, Germans Trias i Pujol University Hospital, Badalona, Spain; High Content Genomics and Bioinformatics Unit, Germans Trias i Pujol Research Institute (IGTP), Badalona, Spain; Inflammatory Bowel Diseases Research Group, Germans Trias i Pujol Research Institute (IGTP), Badalona, Spain; Biomedical Research Network Center for Hepatic and Digestive Diseases (CIBEREHD), Badalona, Spain; Department of Gastroenterology, Germans Trias i Pujol University Hospital, Badalona, Spain; Biomedical Research Network Center for Hepatic and Digestive Diseases (CIBEREHD), Badalona, Spain; Translational Research in Hepatic Diseases Group, Germans Trias i Pujol Research Institute (IGTP), Badalona, Spain; Inflammatory Bowel Diseases Research Group, Germans Trias i Pujol Research Institute (IGTP), Badalona, Spain; Biomedical Research Network Center for Hepatic and Digestive Diseases (CIBEREHD), Badalona, Spain; University Hospital Joan XXIII, Pere Virgili Health Research Institute (IISPV), Rovira i Virgili University, Tarragona, Spain; Biomedical Research Network Center for Hepatic and Digestive Diseases (CIBEREHD), Badalona, Spain

**Keywords:** Crohn disease, creeping fat, TNBS-induced colitis

## Abstract

**Background:**

Creeping fat (CrF) has emerged as a key pathological feature of Crohn disease (CD). Available data suggest that microbial translocation in CD may trigger CrF development, potentially exacerbating intestinal inflammation and disrupting homeostasis. However, the role of CrF in disease progression remains poorly understood, raising the need for experimental models.

**Methods:**

Colitis was induced using 2,4,6-trinitrobenzenesulfonic acid (TNBS) in Sprague-Dawley rats kept in conventional housing conditions. Colonoscopy and weight follow-up observations were performed 3 and 5 days after colitis induction. Samples were collected at day 5 for histopathological staining and cytokine gene expression analyses.

**Results:**

Mesocolic adipose hyperplasia resembling CrF-like mesentery was present in both male and female TNBS-treated rats, with no significant sex-related variation in prevalence. Endoscopic evaluation revealed that only TNBS-treated rats with a colonoscopic score greater than 7 (out of 9) exhibited a significant presence of a CrF-like mesentery. Furthermore, a strong correlation was observed between the severity of colonic inflammation and the presence of CrF-like mesentery, including hyperplastic adipocytes, increased immune cell infiltration, and fibrosis. Molecular characterization showed an upregulation of key inflammatory cytokines—interleukin (IL)-1β, IL-6, and tumor necrosis factor (TNF)-α—and the pathogen-recognition receptors—Toll-like receptor 2 (TLR2) and nucleotide-binding oligomerization domain–containing protein 2 (NOD2)—in the CrF-like mesocolon, as observed in human CrF. Finally, animals exhibiting CrF-like mesocolon showed a translocation of Gram-positive cocci in the subserosal layer.

**Conclusions:**

Mesocolic hyperplasia, closely replicating the key histopathological and molecular features of CD-related CrF, developed in half of the rats in this model. This model provides a cost-effective platform for studying the interplay between intestinal inflammation and mesenteric adipose tissue remodeling.

Conference presentations:

- 19th Congress of European Crohn’s and Colitis Organisation, ECCO 2024, 21–24 February. E-poster *Journal of Crohn’s and Colitis*, Volume 18, Issue Supplement_1, January 2024, Pages i272–i273. https://doi.org/10.1093/ecco-jcc/jjad212.0152
- 20th Congress of European Crohn’s and Colitis Organisation, ECCO 2025, 19–22 February. E-poster. *Journal of Crohn’s and Colitis*, Colitis, Volume 18, Issue Supplement_1, January 2025, Pages i568–i568. https://doi.org/10.1093/ecco-jcc/jjae190.0344

Key Messages

*What is already known?*

TNBS-induced colitis models have been extensively used in the study of the pathophysiology of Crohn disease. Creeping fat (CrF) stands as an important hallmark of the disease, affecting its severity. To date, no in vivo model has successfully replicated the development of CrF.

*What is new here?*

We present a TNBS-colitis rat model in which, under conventional conditions, at least half of the animals presented hyperplastic mesocolic adipose tissue related to bacterial translocation and transmural inflammation.

*How can this study help patient care?*

This animal model can help increase understanding of the role of creeping fat in the severity of Crohn disease in addition to being a new tool for testing new therapeutic and surgical strategies.

## Introduction

Crohn disease (CD) is a chronic inflammatory bowel disease (IBD) characterized by persistent inflammation in the gastrointestinal tract, leading to complications such as fibrosis, strictures, intestinal fistulae, and intraabdominal abscesses; moreover, even when the involved gut is surgically removed, the disease tends to develop once again.^1^ Among the hallmarks of CD, creeping fat (CrF) has gained increasing interest as a key pathological feature.^2^ CrF is defined as the hyperplastic expansion of mesenteric adipose tissue surrounding the inflamed intestinal segments, and its role in disease progression and recurrence remains poorly understood. Emerging evidence suggests that microbial translocation in CD may act as a central trigger for CrF development, providing new insights into the pathophysiology of the disease.^3^^,^^4^

Recent studies have suggested that CrF may contribute to intestinal inflammation by acting as a secondary inflammatory source, disrupting intestinal homeostasis, and exacerbating disease severity.^5^^,^^6^ Clinically, the surgical removal of CrF as part of an ileocecal resection has been proposed as a potential strategy to improve postoperative outcomes in CD patients.^7^ However, conflicting results have been reported, with some studies supporting the beneficial effects of surgical removal while others reporting no significant impact with this procedure.^7^^,^^8^ This controversy highlights the need for appropriate experimental models to investigate the mechanisms underlying CrF development and its contribution to CD pathophysiology.

To address this unmet need, in the present study we aimed to establish and characterize an experimental model of colitis, induced by 2,4,6-trinitrobenzenesulfonic acid (TNBS) in rats, which, under specific conditions, led to the development of alterations in the mesocolic adipose tissue closely resembling the changes that transform perilesional mesenteric fat—and are frequently observed in the ileocecal region—into CrF in human CD. Our objective was to provide a novel preclinical platform for investigating the pathological transformation of mesenteric adipose tissue into CrF in the context of intestinal inflammation.

## Material and methods

### Ethical considerations

The animal studies used in this investigation were approved by the Comparative Medicine and Bioimage Centre (CMCiB) of the Catalonia Animal Experimentation Ethical Committee and the competent authorities (Generalitat de Catalunya); experimental project (authorization no. 12610) in strict accordance with European regulations (The European Commission). Commission regulation (EU) No 2019/1390 of July 31, 2019. *Official Journal of the European Union*. 2019:Sep 26;62.) and strict animal welfare standards, following the principles of the 3Rs in research with animal models. A daily supervision protocol was put in place, and specific considerations were assessed: body weight, the external aspect of the animals, response to stimuli, and important aspects of feces. Animal laboratory technicians were trained to recognize the relevant clinical signs, and a veterinarian was responsible for the supervision of the protocol.

### Rats and housing

The experiment was carried out in the CMCiB facilities, which are part of the Germans Trias i Pujol Research Institute (IGTP). Ten 7-week-old Sprague-Dawley rats (50% females) (Sprague Dawley SD; Envigo, Barcelona, Spain) were housed in filtered top polycarbonate cages. Animals were housed in groups of two and 3 rats per cage, at a constant temperature of 20°-24 °C, 60% humidity, with 20 air renewals per hour and 12-hour light/darkness cycles. Cellulose paper and cardboard rolls were introduced as elements of environmental and social enrichment.

### TNBS induced colitis model

**Figure 1.**
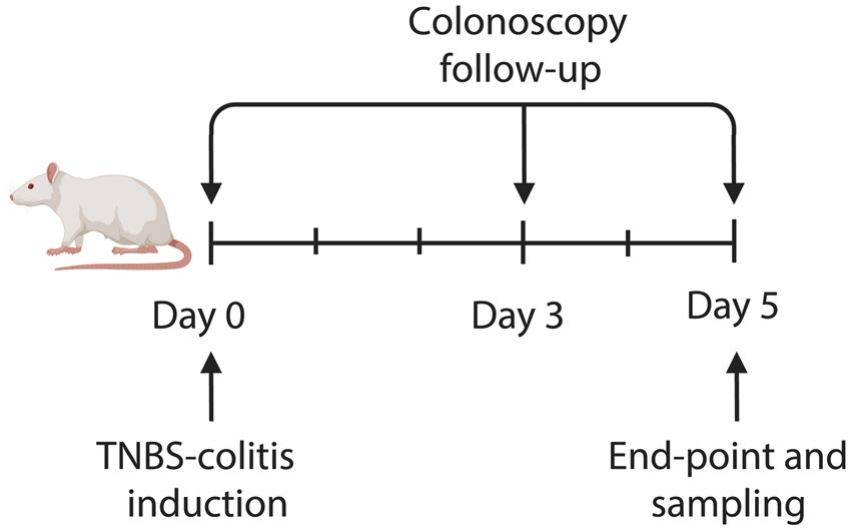
Graphical representation of the experimental design. Time is shown in days. Colitis TNBS administration, colonoscopy follow-up and the study endpoint are indicated by arrows or brackets. The endpoint corresponds to the last day of the trial. TNBS, 2,4,6-trinitrobenzene sulfonic acid.

After 1 week of acclimation, animals underwent experimental colitis induced by the intrarectal instillation of 0.25 mL of 30 mg of TNBS (Merck KGaA, Darmstadt, Germany) in 50% ethanol (vol/vol) at day 0 (Figure 1), as previously reported,^9^ according to the method described by Morris et al.^10^ We also included a group without TNBS induction (sham colitis), consisting of 6 untreated rats (*n* = 3 females and *n* = 3 males). Samples from these animals were exclusively used for the qRT-PCR experiments.

Furthermore, to reinforce the robustness of CrF detection, we examined its frequency in an independent group of 20 rats receiving a single TNBS dose, as well as in 10 rats subjected to 4 repeated weekly TNBS administrations (50% females in both experiments). In this multiple-dose model, we also characterized the histological features of murine CrF and compared them with those observed in the single-dose cohort. Full methodological details and complete results of the multiple administration TNBS colitis model are provided in Figure S1.

### Experimental design and colonoscopy follow-up

The overall experimental designs and timelines of the endoscopic assessments are summarized in Figure 1. Endoscopic evaluations were carried out on day 0 before TNBS-colitis induction and on days 3 and 5 after colitis induction, using an Olympus Video bronchoscope EVIS EXERA II (BF-1T180)-type endoscope with an outer diameter of 6.0 mm. After an 8-hour fasting period with free access to drinking water, rats were anesthetized by isoflurane inhalation and placed in the supine position. Remaining feces were flushed away by injecting warm water through the anus. The endoscope was gently introduced up to the proximal colon until reaching the cecum and the lumen was carefully examined to assess damage to the colonic mucosa and its evolution during the study. Room air was used for insufflation during the endoscopy. The presence, number, and extent of ulcers and their appearance were carefully described, as well as the presence of hyperemia, fibrinous exudate, blood, and colonic stenosis. Based on these findings, an endoscopic severity score (0-9) was used, adapted from Vermeulen W et al.^11^ (Table 1).

**Table 1.** Colonoscopic scoring system for evaluating intestinal lesions.

Score	Hyperemia	Ulcera	Morphology	Depth	Stenosis
**0**	None	None	Discontinuous	Flat	None
**0.5**	Mild	–	–	–	–
**1**	Severe	Small	Continuous	Mild geographic	Mild
**2**		Semicircular		Severe geographic	Severe
**3**		Circular			

### Necropsy and sampling

*Rats* were anesthetized with inhaled isoflurane (induction 4%-5% and maintenance with 1.5%-2%) in oxygen, and a colonoscopy was performed to assess mucosal damage. Subsequently, while the animal remained under general anesthesia, euthanasia was carried out through air embolism by intracardiac puncture. A midline laparotomy allowed complete examination of the abdominal cavity and the entire intestinal tract package, which was resected from the Treitz angle to the anus, along with the omentum, mesentery, and mesocolon. No additional intra-abdominal lesions were detected. The small bowel was separated at the ileocecal valve, and the cecum was removed, yielding the colon together with its mesocolon as the final specimen. The entire colon was longitudinally opened, rinsed, weighed and its total length was measured. Biopsies were collected from regions showing greater macroscopic involvement (transmural involvement and adipose hyperplasia). A sectional piece of affected intestine with adhered mesocolon was preserved in 4% formol for 24 h, embedded in paraffin, and cut in sections of 3-μm of thickness. Sections were stained using hematoxylin-eosin (HE), Masson trichrome (MT), and Gram stains, or used for immunohistochemistry. Part of the affected intestine and the contiguous mesocolon were incubated separately with RNAlater overnight, and then dried and frozen at −80 °C for the subsequent quantitative real-time polymerase chain reaction (qRT-PCR) experiments.

### Histological staining and evaluation

Formalin-fixed, paraffin-embedded (FFPE) tissue sections were stained with HE, MT, and Gram stains according to standard protocols. After completion of all 3 staining protocols, sections were dehydrated and mounted using ProLong TM Glass Antifade Mountant (Thermo Fisher, Barcelona, Spain).

Intestinal lesions were assessed in a blinded fashion, using a semiquantitative modified version of the scores described by Sann et al.^12^ (Table 2), which integrates the main pathological features expected in this TNBS-colitis model, including the extent and intensity of acute and chronic inflammation, crypt damage, mucosal and submucosal oedema, loss of goblet cells, reactive epithelial hyperplasia, and fibrosis—evaluated using MT staining (aniline blue–stained collagen in rat samples and light-green–stained collagen in human samples). The final score was determined by adding the aforementioned features (maximum score 25 points).

**Table 2.** Histopathological scoring of intestinal lesions induced by TNBS administration.

Score	Acute inflammation		Chronic inflammation		Crypt damage	Edema		Loss of goblet cells	Reactive epithelial cell hyperplasia	Fibrosis
	Extent	Intensity	Extent	Intensity		M	SubM			
**0**	None	None	None	None	None	None	None	None	None	None
**1**	M	Low	M	Low	Low	Yes	Yes	Mild	Yes	Low
**2**	SubM	Mild	SubM	Mild	Mild			Severe		Mild
**3**	Musc	Severe	Musc	Severe	Severe					Severe
**4**	TM		TM							

Abbreviations: M, mucosal; Musc, muscularis; SubM, submucosal; TM, transmural; TNBS, 2,4,6-trinitrobenzene sulfonic acid.

Bacterial presence was assessed by using Gram staining. Finally, adipogenesis was evaluated by counting the number of adipocytes in a 1 mm^2^ field in specific hotspots of the mesocolon. All evaluations were conducted with a Nikon Eclipse Ci microscope (Nikon Instruments Inc, United States).

In order to compare the mesocolic hyperplasia observed in rats to the CD CrF, we used 37 paraffin-embedded CrF sections stained for HE and MT from the Biobank at IGTP (ISCIII Biobank National Register code: B.0000643). These samples were collected from active CD patients with complicated CD who underwent a surgical intestinal resection. CrF samples were obtained from the hyperplastic mesenteric adipose tissue matching with the inflamed zone of the intestine. Samples from healthy mesenteric human adipose tissue were also obtained from surgery in patients undergoing low anterior resection of the rectum.

### Immunohistochemistry for myeloperoxidase and CD45

In brief, FFPE sections (3 µm) of colonic wall were deparaffinized, rehydrated, and subjected to heat-induced antigen retrieval in citrate buffer (pH 6.0). After undergoing blocking of endogenous peroxidase (3% H_2_O_2_) and non-specific binding, sections were incubated overnight at 4 °C with anti-myeloperoxidase (MPO; Dako FLEX, Polyclonal rabbit anti-human, Dako Omnis, Agilent, CA, United States) to identify neutrophils and anti-CD45 to detect leukocytes (Dako FLEX, Monoclonal mouse anti-human, clone 2B11 + PD7/26, Dako Omnis, Agilent, CA, United States). Detection was performed using an HRP/DAB (horseradish peroxidase and 3,3'-diaminobenzidine) detection kit (Abcam, Cambridge, United Kingdom), followed by hematoxylin counterstaining. Negative controls omitting the primary antibody were included.

### Quantification of gene expression by qRT-PCR

Around 25 mg of intestine wall and/or mesocolon tissue samples were fragmented using a mortar cooled in liquid nitrogen. Tissue was then homogenized in 700 μL of QIAzol reagent (QIAgen, Madrid, Spain) using a gentleMACS™ tissue dissociator (Miltenyi Biotech, Teterow, Germany) and centrifuged to remove the lipid layer for the mesocolon samples. After chloroform was added, the sample was separated into 3 phases. The upper phase was then used for RNA purification using the miRNeasy Mini kit in the QIAcube system (QIAGEN, Hilden, Germany), following the manufacturer’s protocol. RNA integrity was evaluated by an electrophoresis system Agilent 6000 Nano kit (Agilent Technologies, Santa Clara, CA, USA) using RNA Bioanalyzer chips. Only RNA integrity numbers equal to or higher than 6.5 were deemed good and processed. A total of 1 μg of total RNA, was DNase treated using the TURBO DNase (Thermofisher Scientific, Madrid, Spain) and retrotranscribed to cDNA using the PrimeScriptTM RT reagent (Takara, Shiga, Japan), following the manufacturer’s instructions. TaqMan^®^Assays (ThermoFisher Scientific, Madrid, Spain) for qRT-PCR of *Il1b*, *Il6*, *Il10*, *Il17a*, *Nod2*, *Tgfbr1*, *Tlr2*, *Tlr4*, *Tnfa* (references: Rn00580432_m1, Rn01410330_m1, Rn00563409_m1, Rn01757168_m1, Rn01770864_m1, Rn00562811_m1, Rn01769726_m1, Rn00569848_m1 and Rn99999017_m1, respectively) were used. PCR thermal cycling included initial denaturing at 95 °C for 10 seconds, 40 cycles of 95 °C for 15 seconds, and 60 °C for 1 minute (LightCycler480 system; Roche Diagnostics, Basel, Switzerland). Data from qRT-PCR were calculated using the 2^−ΔCt^ method.^13^ The results obtained were normalized using *Actb* and *Gapdh* gene expression (Rn00667869_m1 and Rn01775763_g1) as housekeeping genes.

### Statistical analysis

Statistical analyses were conducted to compare data between 2 groups: animals presenting mesocolic adipose tissue hyperplasia and those without. Sample size was determined based on prior experience with this model and in accordance with the 3Rs principles (Replacement, Reduction, Refinement) to minimize animal use and promote ethical research in this study. Data normality was assessed using the Shapiro-Wilk test. As several variables did not meet normality assumptions, the non-parametric Mann-Whitney U test was applied for independent continuous variables, while the Chi-square test was used for categorical variables. All analyses were performed using R Studio (R version 4.2.2).

## Results

### Induction of hyperplastic mesocolic adipose tissue by TNBS-induced colitis in rats

**Figure 2.**
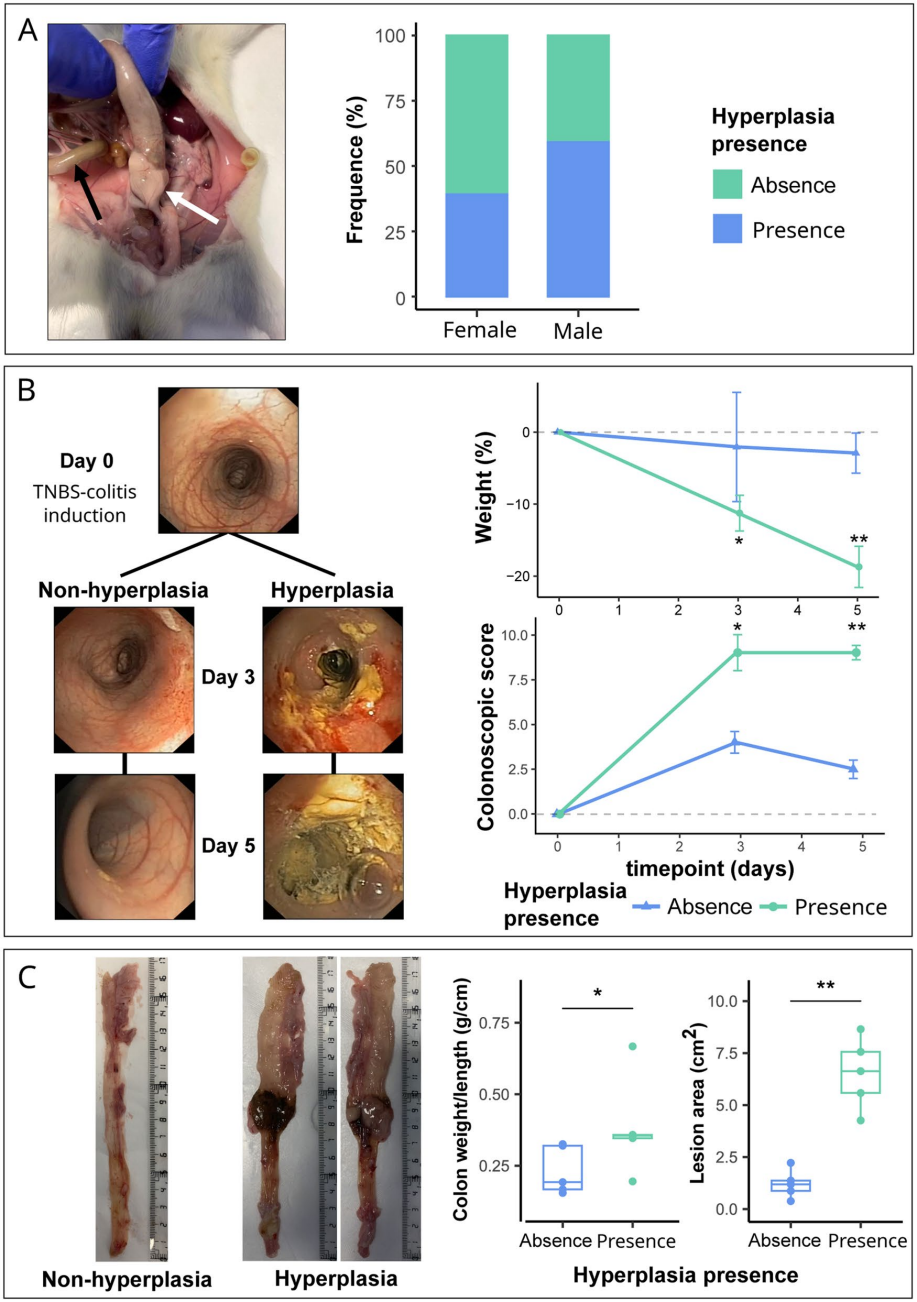
Follow-up of TNBS-colitis severity and macroscopic evaluations, with a focus on mesocolic adipose tissue hyperplasia. (A) Macroscopic images of the mesocolon showing hyperplasia, with a histogram illustrating the frequency of hyperplasia after TNBS-colitis induction, categorized by the sex of the animal. White arrow indicates mesocolic adipose tissue hyperplasia and a black arrow indicates normal mesentery. (B) Colonoscopy and weight follow-up to assess the severity of the TNBS-colitis. Colonoscopy images represent animals from both groups: the presence of mesocolic adipose tissue hyperplasia and its absence. The severity of the lesion observed in the colonoscopy is pictured as a numerical score on the *x*-axis through time in the *y*-axis. Weight is represented as % of change from day 0 in the *y*-axis through time in the *x*-axis. Asterisks represent significant differences in each day between both groups. (C) Macroscopic evaluations performed during the necropsy. Images of the dissected colon from animals with and without mesocolic hyperplasia. The area of the lesion (cm^2^) and the colon weight-to-length ratio (g/cm) are shown as boxplots with jittered data points. The central line represents the median, the box displays the IQR, and the whiskers indicate the minimum and maximum values. Significance levels: * *P*-value ≤.05; ** *P*-value ≤.01. TNBS, 2,4,6-trinitrobenzenesulfonic acid.

During the in vivo experimental procedure, we observed that 3 out of 5 TNBS-treated male rats and 2 out of 5 TNBS-treated females presented macroscopic CrF-like abnormal growth of the mesocolic adipose tissue in the perilesional region, coincident with stenotic areas (Figure 2A). The frequency of mesocolic adipose tissue hyperplasia was consistent across independent TNBS experiments. In the main single-dose TNBS cohort, 50% of animals (5/10) developed CrF-like hyperplasia with no sex-related differences. This incidence was comparable to that observed in an independent validation experiment: 57.89% (11/19); as well as in a repeated-dose TNBS protocol: 60% (6/10). Notably, the macroscopic and histological characteristics of the hyperplastic mesocolon in the multi-dose TNBS model were virtually identical to those observed after a single dose, showing similar adipocyte hyperplasia, inflammatory cell infiltration, and fiber deposition (Figure S1B-C). Together, these findings demonstrate the robustness and reproducibility of CrF-like changes across experimental settings.

### Correlation of colitis severity with mesocolic adipose tissue hyperplasia in TNBS colitis

Baseline colonoscopy (day 0) revealed no lesions (Figure 2B). By day 3, all animals had developed colonic lesions, exhibiting significant inter-animal variability. Four animals presented severe, hyperemic circumferential geographic lesions with exudate and blood (score 9), 4 showed moderate lesions (score 4 or 5), and 2 had minimal small lesions (score 2). By day 5, animals with initially severe lesions (score 9) maintained the same severity. Those with mild lesions improved slightly (from score 2 to 1). The animals with intermediate lesions (scores 4-5) had variable progression: most showed mild improvement (scoring between 2.5 and 3.5), although in 1 animal the score worsened to a score of 7. Furthermore, the presence of mesocolic hyperplasia was associates with animals scoring 7 or greater at both days 3 and 5 (day 3, *P = .029;* day 5, *P = .009*), with no observed differences between sexes.

The mice in the group showing mesocolic hyperplasia had higher weight loss and, additionally, the ulcerated area and colon weight-to-length ratio further supported these associations, as both were significantly correlated with mesocolic hyperplasia (Figure 2C; lesion area, *P* = .008; colon weight/length ratio, *P* = .032). Again, no differences were observed between sexes.

**Figure 3.**
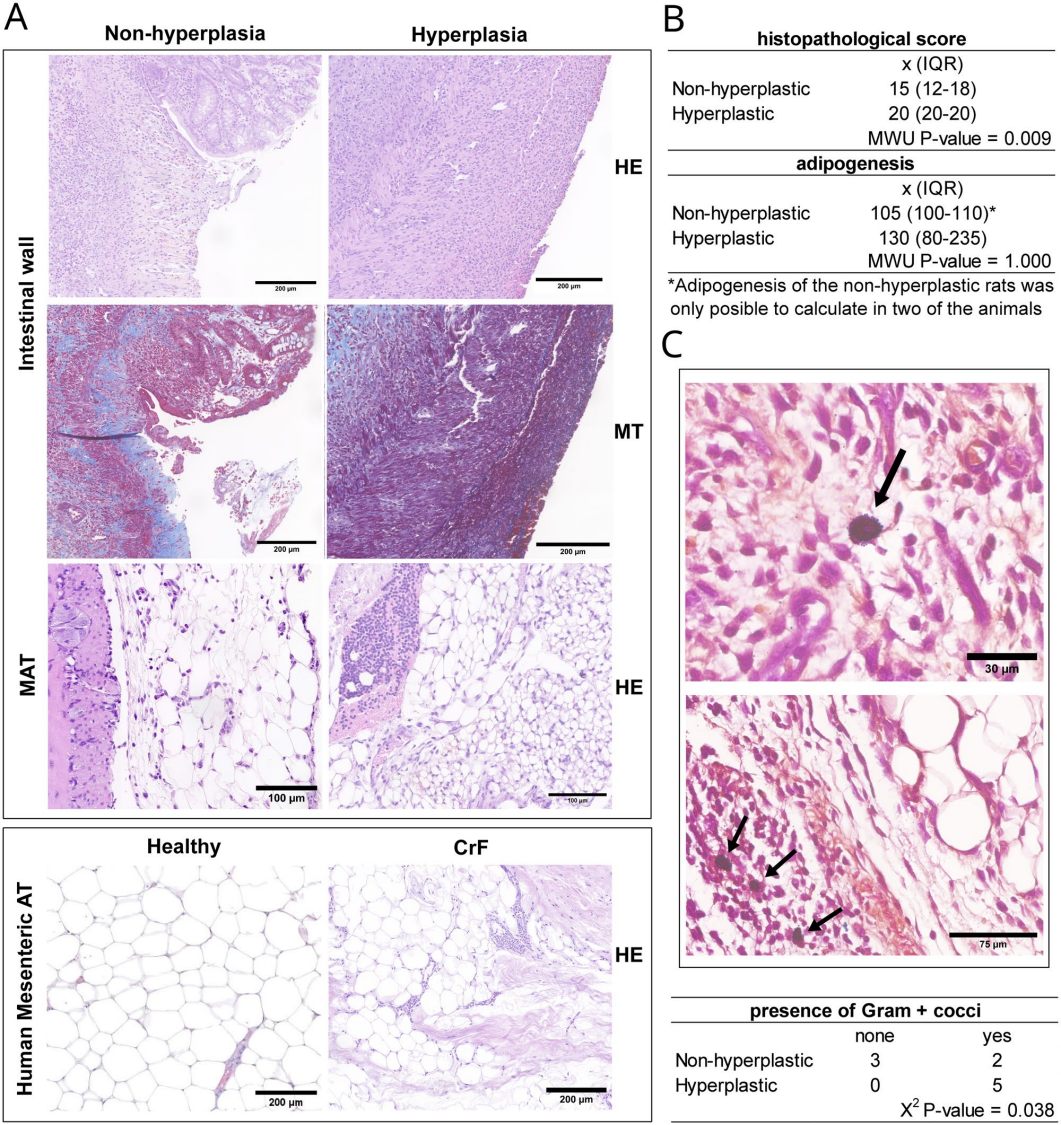
Histopathological evaluation of both intestinal mucosa and MAT. (A) Microscopic images of the intestinal wall and MAT from both animal groups: hyperplastic MAT and non-hyperplastic. The intestinal wall is shown using HE and MT staining, while MAT is presented using HE stains. (B) Statistical analysis of histological scores and adipogenesis, with data presented as median and IQR. (C) Images depicting the presence of Gram^+^ cocci in the subserosa of the animals exhibiting mesocolic hyperplasia. The table with the number of animals showing the presence of translocated bacteria is presented. CrF, creeping fat; HE, hematoxylin-eosin; MAT, mesocolic adipose tissue; MT, Masson’s trichrome; MWT, Mann-Whitney U test.

To further characterize the intestinal and mesocolic changes associated with TNBS-induced colitis, histopathological evaluations were performed, comparing the intestinal mucosa and mesocolic adipose tissue from those animals with TNBS-induced colitis showing hyperplasia of the adipose tissue with those showing a macroscopically normal mesocolon. Figure 3A shows histological stains of both intestinal mucosa and mesocolic adipose tissue in both groups. The final histopathological score of the intestinal mucosa of those animals showing mesocolic hyperplasia was significantly higher than that of those animals without adipose hyperplasia (*P* = .009) (Figure 3B). Moreover, the degree of the acute inflammation was significantly more frequently severe in those animals with mesocolic hyperplasia (*P* = .009) (Table S1). The chronic inflammation was similar in both groups. Cryptitis was also more intense and severe in those animals with hyperplasia (*P* = .036). Moreover, neutrophilic infiltration, although not significant, reached a transmural level surpassing the level in the *muscularis propria* in 4 out of the 5 mice showing adipose hyperplasia with a median score of 4, which was higher than scored in those without mesocolic hyperplasia (x͂ = 2). Interestingly, the extent and severity of fibrosis showed a non-significant higher trend (*P* = .073). Additionally, fiber deposition seemed to be implicated in wound healing in those rats without hyperplasia whereas, in those animals with hyperplasia, fibers were still unstructured (Figure 3A). Fiber deposition in the adipose tissue was also observed in those animals with hyperplasia (Figure S2). No differences were observed regarding the presence of oedema in the mucosa or submucosa, and it was present in almost all the animals. None of these evaluations differed between sexes (Table S2).

Semi-quantitative image analysis of immunohistochemical staining revealed that the MPO^+^ area was significantly greater in hyperplastic animals 2.12 ± 0.55 MPO^+^ staining (%), compared with non-hyperplastic animals 0.73 ± 0.19 MPO^+^ staining (%), indicating increased acute neutrophilic infiltration (Figure S3A). Similarly, CD45+ staining was markedly elevated in the hyperplasia group (3.63 ± 470.85% CD45+ staining), consistent with enhanced chronic inflammatory infiltrates, relative to the non-hyperplasia group 1.10 ± 0.16 CD45^+^ staining (%), (Figure S3B). Morphometric analysis of adipocytes demonstrated a larger cross-sectional area in hyperplastic regions 1762.51 ± 47.42 µm^2^ compared to non-hyperplastic tissue 3940.46 ± 140.53 µm^2^, reflecting adipocyte hyperplasia associated with mesocolic growth (Figure S3C). These results collectively highlight the pronounced inflammatory and morphological changes occurring in the mesocolic adipose tissue of animals with adipose hyperplasia.

**Figure 4.**
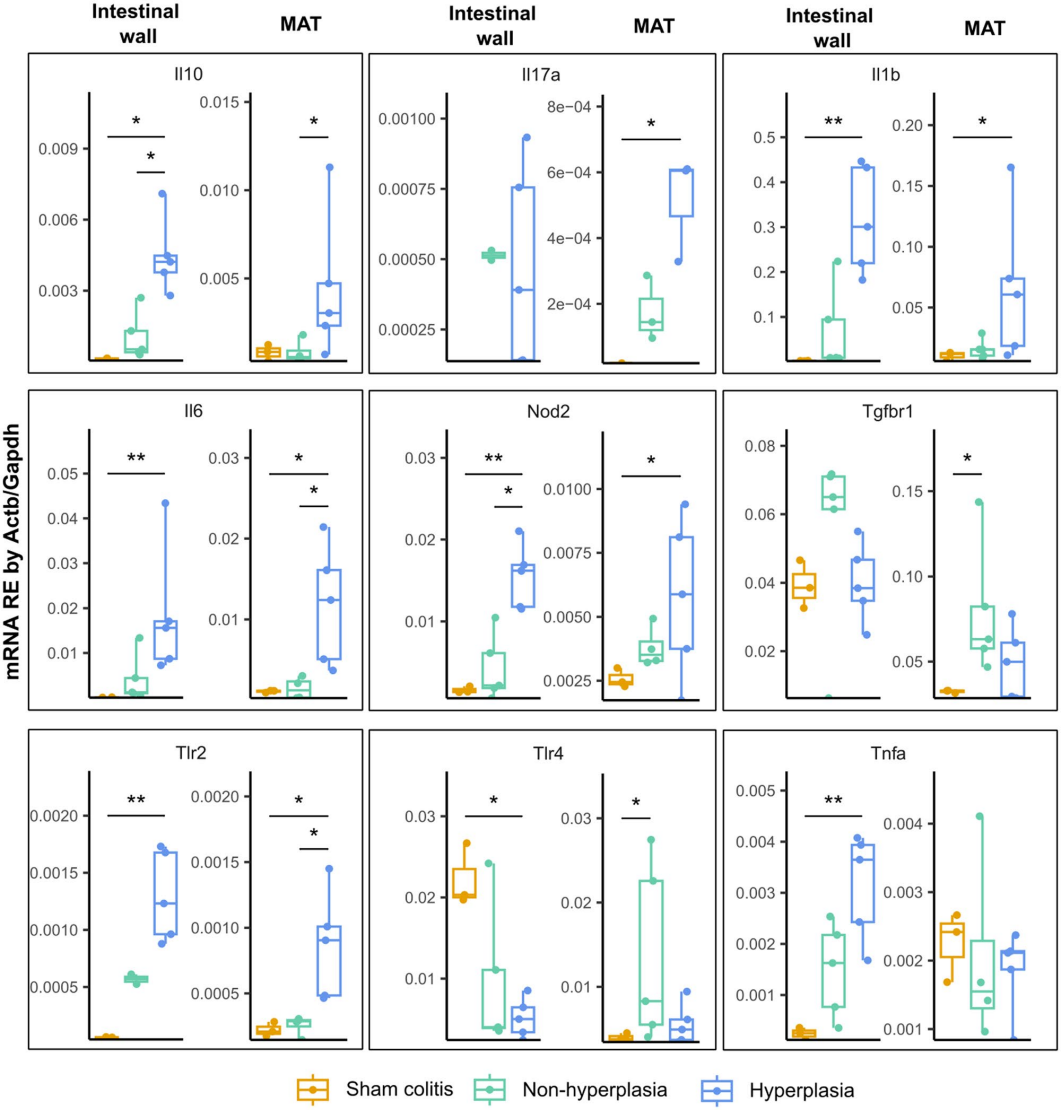
Expression of the inflammatory markers in both intestinal mucosa and MAT. qRT-PCR quantification of mRNA presented as RE. Data are represented as points summarized in boxplots, indicating IQR, and bars, indicating maximum and minimum. The median point is depicted as a bar inside the box. Treatment groups are differentiated using colors. Asterisks indicate significant differences between groups, as determined by the Kruskal-Wallis test with post-hoc Dunn’s test. Significance levels are: *P-value ≤.05; **P-value ≤.01. MAT, mesocolic adipose tissue; qRT-PCR, quantitative real-time polymerase chain reaction; RE, relative expression.

Furthermore, qRT-PCR experiments were performed in both the mesocolon and the inflamed mucosa, to assess inflammatory status. The main cytokines and pattern-recognition receptors (PRRs) involved in inflammation and bacterial detection were analyzed. Regarding the results in the intestinal mucosa, the expression of interleukin 1 beta gene (*Il1b*), interleukin 6 gene (*Il6*), interleukin 10 gene (*Il10*), tumor necrosis factor alpha gene (*Tnfa*), toll-like receptor 2 gene (*Tlr2*), and nucleotide-binding oligomerization domain containing 2 gene (*Nod2*) was increased in those rats showing mesocolic hyperplasia compared to the sham colitis group, while there was no increase with regard to the animals not showing mesocolic hyperplasia (Figure 4). Importantly, *Il10* and *Nod2* were upregulated in the hyperplastic mesocolon group when compared to the non-hyperplastic mesocolon group.

The hyperplastic mesocolon showed elevated levels of *Il1b*, *Il6*, *Il17a*, *Tlr2*, and *Nod2* when compared to the sham colitis group whereas this difference was not present in animals not showing hyperplasia. The most important differences were observed in *Il6*, *Il10* and *Tlr2*, which were upregulated in hyperplastic mesocolic adipose tissue compared to non-hyperplastic tissue. Of note, the expression level of the TGF-β receptor gene *Tgfbr1* was significantly upregulated in the group that did not present with hyperplasia. Finally, there was an increase of *Tlr4* expression in both tissues in those animals without mesocolic adipose hyperplasia.

These observations coincide with the results that our research group observed from the analysis of human CrF obtained from the intestinal resection of CD patients with disease that was complicated and/or non-responsive to biological treatments, where the protein presence of the cytokines IL-1β, IL-6, IL-10, and TNF-ɑ was higher than in the mesenteric adipose tissue of severely obese patients.^14^

Finally, Gram^+^ cocci colonies were frequently detected in the subserosa, with 7 out of 10 animals exhibiting these bacterial clusters (Figure 3C). Notably, all animals with mesocolic hyperplasia displayed subserosal Gram^+^ cocci colonies, while only 2 animals without hyperplasia did so (*P* = .038).

### Comparison of rat mesocolic adipose tissue hyperplasia in TNBS colitis with CrF in Crohn disease

Rat hyperplasia adipose tissue stainings were compared to those of human CrF from CD patients (Figure 3A). Some similarities were found between human CrF and rat hyperplastic adipose tissues. First, higher adipogenesis was observed together with marked immune cell infiltration in both groups when compared with non-hyperplastic adipose tissue from rats not showing CrF-like features and exhibiting healthy mesenteric adipose tissue higher adipogenesis was observed together with marked immune cells infiltrate in both groups when compared to non-hyperplastic adipose tissue from rats not showing CrF-like features and healthy mesenteric adipose tissue. Furthermore, fiber deposition was found in both rat and human hyperplastic adipose tissues (Figure S2).

## Discussion

Our findings provide compelling evidence that the TNBS-induced colitis model in rats can serve as a valuable experimental tool for investigating CrF, a hallmark feature of CD. Despite its extensive use for over 3 decades to study intestinal inflammation,^9^^,^^15^ this model has not previously been explored in the context of profound alterations of mesocolic adipose tissue architecture. Here, we have shown that TNBS colitis induces a reproducible hyperplastic mesocolic response that closely mirrors human CrF at the macroscopic, histological, immunological, and transcriptional levels.

A central observation of this study was that CrF-like mesocolic hyperplasia developed in approximately half of TNBS-treated rats. The incidence was 50% in the main single-dose cohort, 57.9% in an independent validation experiment, and 60% in rats treated with a repeated-dose TNBS protocol. These similar frequencies across independent experiments support the robustness of CrF-like induction in this model. Importantly, the morphological and histological phenotype of the CrF-like hyperplasia was nearly identical in both protocols, including adipocyte hyperplasia, immune-cell infiltration, and fiber deposition, confirming that single-dose TNBS colitis is sufficient to trigger the full CrF-like phenotype. No differences between males and females were observed in any experiment.

The TNBS model recapitulates several traits of chronicity associated with chronic intestinal inflammation, even though it is classically defined as an acute model.^16–18^ Of particular relevance is the ability of this model to induce transmural lesions, a feature that distinguishes CD from ulcerative colitis and is not produced in dextran sulfate sodium (DSS)-induced colitis.^19^^,^^20^ Transmural lesions facilitate bacterial translocation and sustained immune activation, thereby promoting CrF development.^21^ In agreement with this finding, animals that developed CrF-like mesocolic hyperplasia consistently exhibited more severe colonic lesions, deeper inflammatory infiltration, and higher histopathological scores, supporting a tight link between inflammatory severity and mesocolic remodeling. In addition, TNBS colitis is associated with a proinflammatory cytokine profile that induced Th17 responses and elevated IL-6, both crucial mediators of chronic intestinal inflammation.^22^^,^^23^

A key finding of our study is the strong association between the severity of TNBS-induced colitis and the presence of hyperplastic mesocolic adipose tissue. Rats exhibiting severe colonic lesions were the ones developing these CrF-like features, characterized by smaller active hyperplastic adipocytes, increased inflammation, and bacterial infiltration in the subserosal region. Histopathological analyses confirmed that colonic acute inflammation in these animals extended deeper into the intestinal wall, reaching a transmural level, a feature consistent with CD pathology.^1^ Immunohistochemistry supports this interpretation: the hyperplastic mesocolon group showed predominantly chronic inflammatory infiltrates, with acute infiltration mainly confined to the mucosa, whereas non-hyperplastic cases exhibited weaker inflammation restricted to the mucosal layer.

Remarkably, this model reproduces several hallmark characteristics of human CrF,^2^ including adipose tissue expansion around intestinal lesions and a significant infiltration of macrophages, neutrophils, and other immune cells, as well as fibrotic processes resembling those observed in patients with CD. The correlation between hyperplastic adipose tissue and increased cryptitis, along with a trend toward enhanced fibrosis, further supports the relevance of this model for investigating the pathogenic mechanisms driving CrF formation in CD.

Consistent with these findings, we observed a significant upregulation of the mRNA expression of key inflammatory cytokines, including IL-1β, IL-6, and TNF-α, as well as PRRs such as TLR2 and NOD2, in both the intestinal mucosa and mesocolic adipose tissue of animals exhibiting CrF-like features. Nevertheless, *Tlr4* was decreased in hyperplastic mesocolic adipose tissue or unaltered in the mucosa of these animals. Notably, *Il10* and *Nod2* were significantly elevated in the hyperplastic mesocolon compared to non-hyperplastic mesocolic tissue, suggesting a potential immunomodulatory response that contrasts with the possible immunoregulatory role of TGF-β in rats without hyperplasia. These findings parallel observations in CrF obtained from CD patients, in whom increased levels of IL-1β, IL-6, IL-10, and TNF-α have been reported.^24^ This molecular similarity corroborates the translational relevance of our model for studying mesenteric immune inflammation in CD.

Unexpectedly, we observed Gram^+^ cocci colonies in the subserosal region of hyperplastic mesocolon, which were significantly more frequent than those in animals without adipose hyperplasia. This observation suggests that bacterial translocation may contribute to mesocolic inflammation and aligns with previous studies implicating microbial dysbiosis and bacterial invasion in CrF pathogenesis in CD.^3^^,^^4^ Identifying these bacteria would be of interest for further investigation, ideally through metagenomic approaches, opening new avenues for preventive or microbiome-modulating strategies in CD.

The presence of such bacteria could also help explain the observed increase in *Tlr2* in hyperplastic animals that, together with the predominance of TLR2-expressing macrophages in CrF,^3^ suggests a key role for innate immunity in orchestrating the response to bacterial translocation. Human mesenteric adipocytes also express multiple PRRs including TLR2, TLR4, NOD1, and NOD2, confirming their ability to respond directly to bacterial challenge.^25^ Nevertheless, causality cannot be established, and further studies—such as macrophage phenotyping, spatial transcriptomics, or microbiota analyses—are needed to clarify the underlying mechanisms.

In particular, to the best of our knowledge, no previously published model has demonstrated that intestinal inflammation alone—without the need for additional bacterial triggers or genetic modifications—is sufficient to induce CrF-like features. Most existing approaches focus on CrF formation because of bacterial components, often using genetically modified mice or models in which bacterial infection facilitates the development of mesenteric fat alterations, ultimately leading to intestinal lesions.^3^^,^^26^ In contrast, our study provides a unique perspective by showing that TNBS-induced inflammation in rats can spontaneously drive CrF-like changes, reinforcing the translational relevance of this model.

Despite these strengths, our study has certain limitations. First, adipose hyperplasia observed in our rat model developed in the mesocolon, whereas in patients with CD the CrF originates in mesentery surrounding the perilesional ileocecal region; however, both structures share key embryonic origin and functions, likely supporting comparable adipose and immune responses.^27^ Secondly, a key limitation is that CrF-like adipose hyperplasia cannot be determined prior to surgery. However, animals exhibiting severe colonoscopic lesions with geographic ulcers (score ≥7) invariably develop CrF-like adipose hyperplasia, allowing 100% accurate prediction.

Overall, our findings establish the TNBS-induced rat model as a cost-effective tool for studying the interplay between intestinal inflammation and mesenteric adipose remodeling in CD. It reproduces transmural lesions and CrF-like adipose changes, recapitulating key histopathological and molecular features of the disease. This model thus provides a reproducible platform to investigate CrF mechanisms and to test potential therapeutic interventions targeting mesenteric inflammation.

## Supplementary Material

izaf328_Supplementary_Data

## Data Availability

Data that support the findings of this study are available from the corresponding author upon reasonable request.
