# Supplementary material for: Characterization of mesocolic adipose hyperplasia in a rat 2,4,6-trinitrobenzenesulfonic acid colitis model and comparison to creeping fat in Crohn disease
Source: Inflamm Bowel Dis. 2026 Feb 18;32(4):610–9. doi: 10.1093/ibd/izaf328 (PMC13220047; doi:10.1093/ibd/izaf328)
Supplement: izaf328_Supplementary_Data [file izaf328_supplementary_data.zip › Supplementary materials.docx]

**Supplementary Tables**

| **Hyperplasia** | **acute inflammation depth** | | | | | | **edema mucosa** | | |
| --- | --- | --- | --- | --- | --- | --- | --- | --- | --- |
|  | none | mucosa | submucosa | muscularis | transmural |  | none | yes |  |
| Absense | 0 | 2 | 1 | 1 | 1 | χ^2^ P-value = 0.187 | 4 | 1 | χ^2^ P-value = 0.292 |
| Presence | 0 | 0 | 0 | 1 | 4 |  | 5 | 0 |  |
|  | **acute inflammation intensity** | | | | | | **edema submucosa** | | |
|  | none | mild | moderated | severe |  |  | none | yes |  |
| Absense | 0 | 4 | 0 | 1 |  | χ^2^ P-value = 0.009 | 0 | 4 | χ^2^ P-value =  1.000 |
| Presence | 0 | 0 | 0 | 5 |  |  | 0 | 5 |  |
|  | **chronic inflammation depth** | | | | | | **reactive epithelial cells hyperplasia** | | |
|  | none | mucosa | submucosa | muscularis | transmural |  | none | yes |  |
| Absense | 0 | 1 | 0 | 0 | 4 | χ^2^ P-value = 0.292 | 0 | 4 | χ^2^ P-value =  1.000 |
| Presence | 0 | 0 | 0 | 0 | 5 |  | 0 | 5 |  |
|  | **chronic inflammation intensity** | | | | | |  | |  |
|  | none | mild | moderate | severe |  |  |  |  |  |
| Absense | 0 | 4 | 1 | 0 |  | χ^2^ P-value = 0.490 |  |  |  |
| Presence | 0 | 3 | 2 | 0 |  |  |  |  |  |
|  | **crypt damage** | | | | | |  |  |  |
|  | mild | moderated | severe |  |  |  |  |  |  |
| Absense | 3 | 1 | 1 |  |  | χ^2^ P-value = 0.036 |  |  |  |
| Presence | 0 | 0 | 5 |  |  |  |  |  |  |
|  | **loss of Goblet cells** | | | | | |  |  |  |
|  | none | mild | severe |  |  |  |  |  |  |
| Absense | 0 | 2 | 3 |  |  | χ^2^ P-value = 0.221 |  |  |  |
| Presence | 0 | 0 | 5 |  |  |  |  |  |  |
|  | **intestinal fibrosis** | | | | | |  |  |  |
|  | none | mild | moderate | severe |  |  |  |  |  |
| Absense | 0 | 2 | 2 | 0 |  | χ^2^ P-value = 0.073 |  |  |  |
| Presence | 0 | 0 | 5 | 0 |  |  |  |  |  |

***Supplementary table 1****. Statistical comparison of histopathological evaluations depending on the presence of adipose tissue hyperplasia.*

***Supplementary table 2****. Statistical comparison of histopathological evaluations depending on the seχ.*

|  | **acute inflammation depth** | | | | | | **edema mucosa** | | |
| --- | --- | --- | --- | --- | --- | --- | --- | --- | --- |
|  | none | mucosa | submucosa | muscularis | transmural |  | none | yes |  |
| Female | 0 | 0 | 0 | 0 | 5 | χ^2^ P-value = 0.158 | 4 | 1 | χ^2^ P-value = 0.292 |
| Male | 0 | 1 | 0 | 0 | 4 |  | 5 | 0 |  |
|  | **acute inflammation intensity** | | | | | | **edema submucosa** | | |
|  | none | mild | moderate | severe |  |  | none | yes |  |
| Female | 0 | 3 | 2 | 0 |  | χ^2^ P-value = 1.000 | 0 | 5 | χ^2^ P-value =  1.000 |
| Male | 0 | 4 | 1 | 0 |  |  | 0 | 4 |  |
|  | **chronic inflammation depth** | | | | | | **epithelial hyperplasia** | | |
|  | none | mucosa | submucosa | muscularis | transmural |  | none | yes |  |
| Female | 0 | 0 | 1 | 2 | 2 | χ^2^ P-value = 0.292 | 3 | 2 | χ^2^ P-value =  0.114 |
| Male | 0 | 2 | 0 | 0 | 3 |  | 5 | 0 |  |
|  | **chronic inflammation intensity** | | | | | | **presence of Gram + cocci** | | |
|  | none | mild | moderate | severe |  |  | none | yes |  |
| Female | 0 | 2 | 0 | 3 |  | χ^2^ P-value = 0.490 | 1 | 4 | χ^2^ P-value =  0.490 |
| Male | 0 | 2 | 0 | 3 |  |  | 2 | 3 |  |
|  | **crypt damage** | | | | | |  |  |  |
|  | none | mild | moderate | severe |  |  |  |  |  |
| Female | 0 | 1 | 1 | 3 |  | χ^2^ P-value = 0.513 |  |  |  |
| Male | 0 | 2 | 0 | 3 |  |  |  |  |  |
|  | **loss of Goblet cells** | | | | | |  |  |  |
|  | none | mild | severe |  |  |  |  |  |  |
| Female | 0 | 0 | 5 |  |  | χ^2^ P-value = 0.221 |  |  |  |
| Male | 0 | 2 | 3 |  |  |  |  |  |  |
|  | **intestinal fibrosis** | | | | | |  |  |  |
|  | none | mild | moderate | severe |  |  |  |  |  |
| Female | 0 | 2 | 3 | 0 |  | χ^2^ P-value = 0.151 |  |  |  |
| Male | 0 | 0 | 4 | 0 |  |  |  |  |  |

**Supplementary Figures**


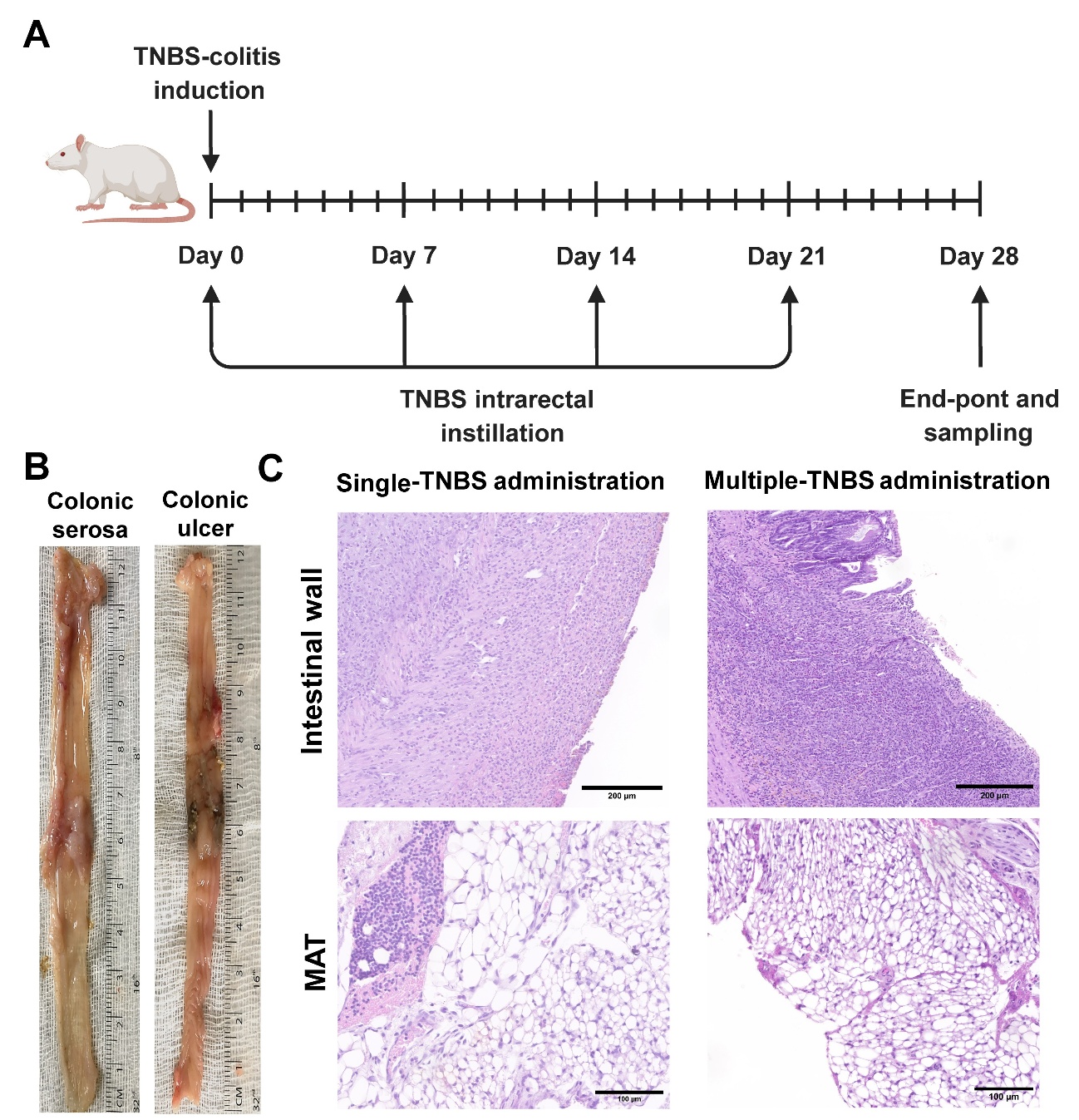


***Supplementary Figure 1.*** *Histopathological evaluation of both intestinal mucosa and MAT in multiple-administration TNBS model.* (A) Graphical representation of the multiple-TNBS administration model experimental design. Multiple-administration TNBS-induced colitis model. Colitis was induced by four rectal instillations of TNBS (0.25 mL of 30 mg in 50% ethanol) on days 0, 7, 14, and 21. Rats were fasted overnight before each induction. (B) Representative macroscopic images of the colon in the multiple-TNBS administration model. (C) Microscopic images of HE staining of the intestinal wall and MAT from both animal groups showing adipose hyperplasia: single-administration and multiple-administration TNBS model. HE, hematoxylin-eosin; MAT, mesocolic adipose tissue; TNBS, 2,4,6-trinitrobenzene sulfonic acid.

**
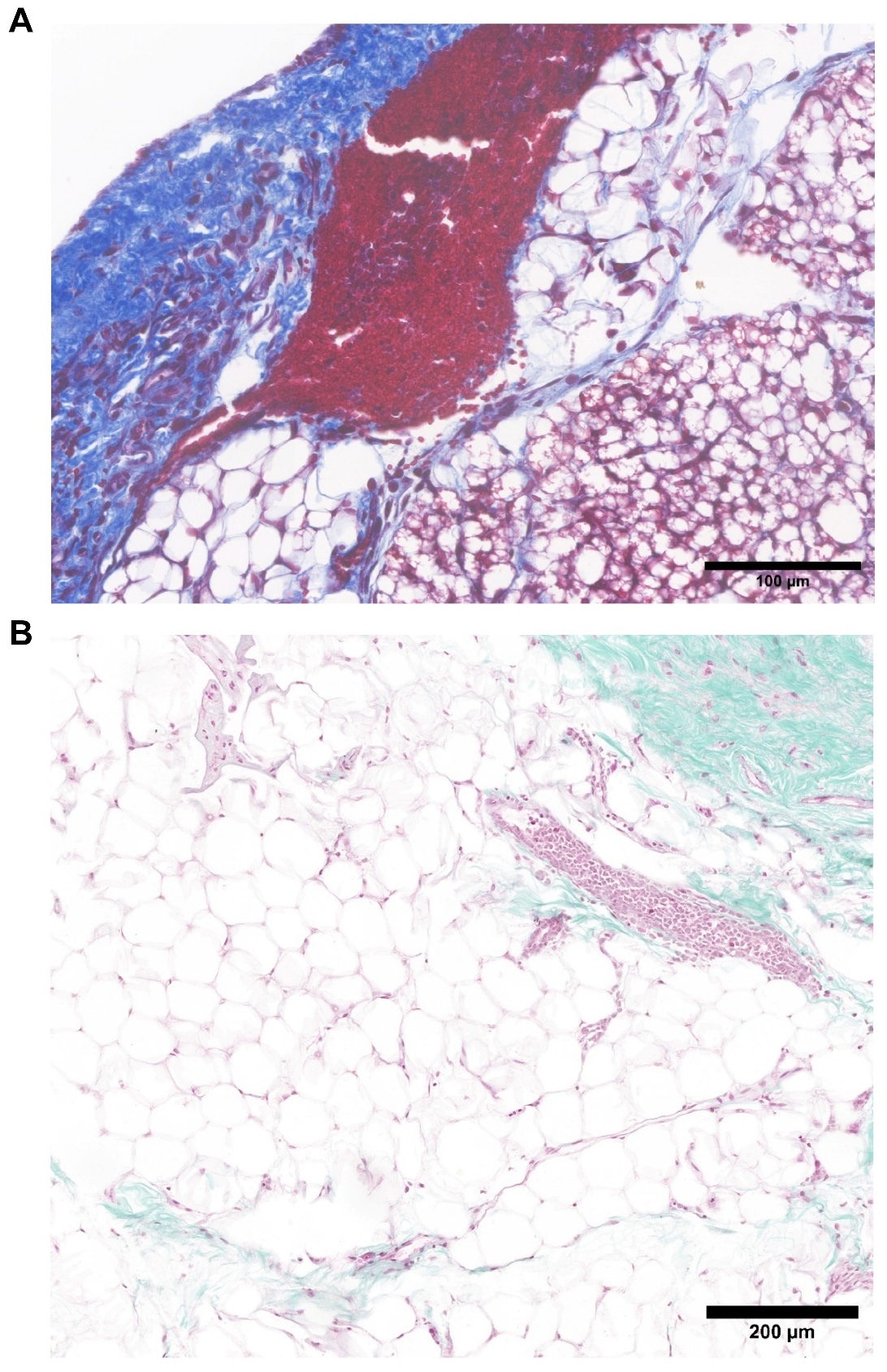
**

***Supplementary Figure 2.*** *Histological images depicting hyperplastic adipose tissue fibrosis.* (A) Mesocolic adipose tissue in the TNBS-induced colitis rat model and (B) in Crohn’s disease creeping fat from human patients. Whole intestinal sections were stained with Masson’s trichrome using aniline blue (rats) or light green (humans) for collagen.

***
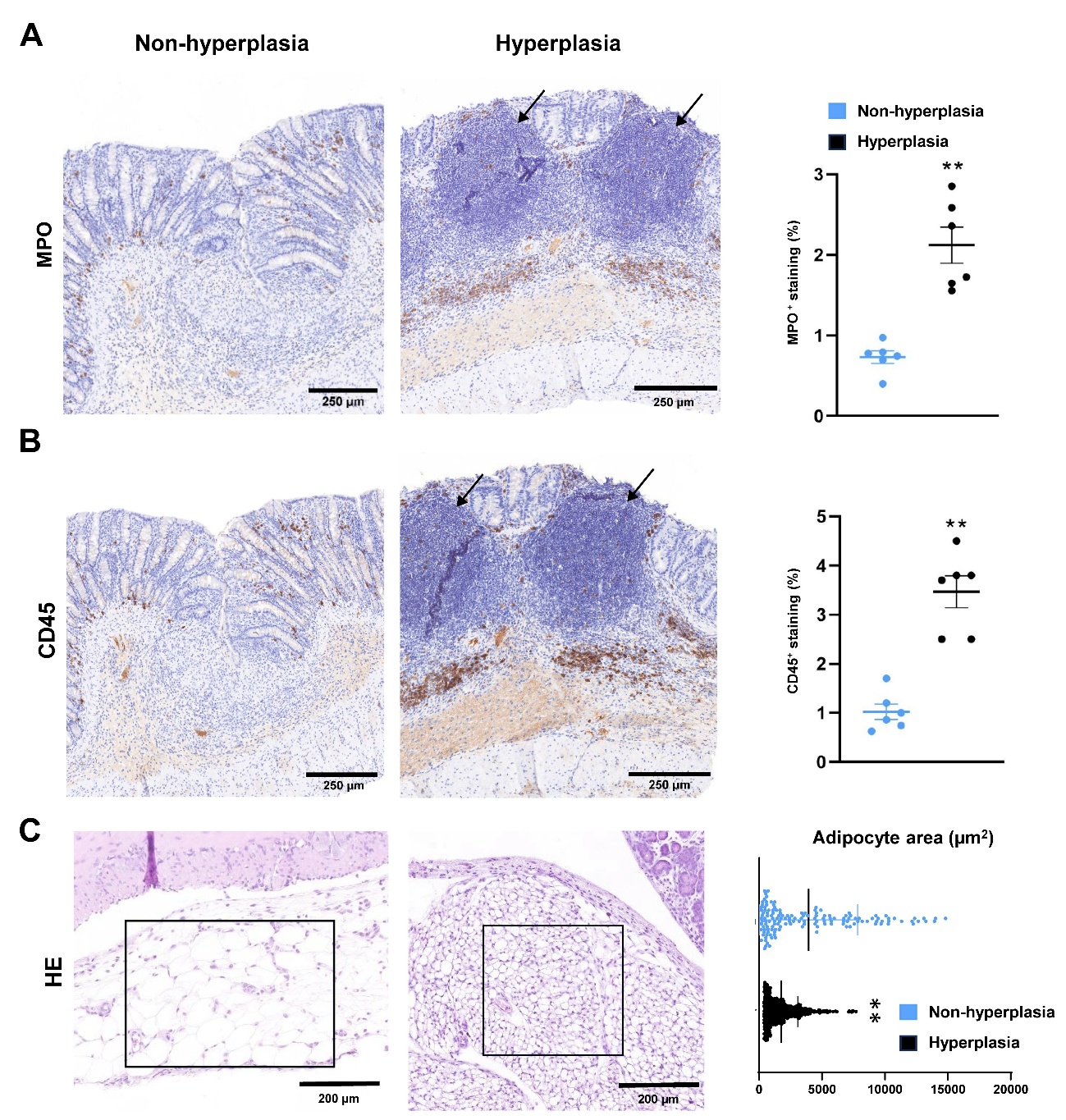
***

***Supplementary Figure 3.*** *Immunohistochemical evaluation of acute and chronic inflammatory infiltrates in TNBS-colitis rats.* Representative sections of MAT and adjacent colon from TNBS-treated rats with and without hyperplasia. (A) MPO staining highlights acute neutrophilic infiltration, more abundant in the mucosa and extending to the submucosa in hyperplastic cases but largely restricted to the mucosa/intraepithelial areas in non-hyperplastic adipose mesocolic tissue. (B) CD45 staining reveals chronic inflammatory infiltrates reaching the submucosa in hyperplastic tissues, while in non-hyperplastic tissues CD45⁺ cells are located superficially, like myeloperoxidase-positive cells. Lymphoid follicle-like neostructures (arrow) appear in hyperplastic mesocolon but are negative for MPO and CD45. (C) HE shows hyperplastic MAT expansion with prominent immune infiltrates. Squares in this image correspond to 1 mm^2^. All the situations evaluated in this figure are accompanied by the corresponding graphs of the semi-quantitative evaluations (MPO, CD45, hyperplasia). Whiskers in graphs represent mean ± SE. *** P ≤ 0.005*. HE, hematoxylin-eosin; MAT, mesocolic adipose tissue; MPO, myeloperoxidase; SE, standard error; TNBS, 2,4,6-trinitrobenzene sulfonic acid.
